# Control of Dielectric and Mechanical Properties of Styrenic Block Copolymer by Graphite Incorporation

**DOI:** 10.3390/ma15217577

**Published:** 2022-10-28

**Authors:** Florin Ciuprina, Denis Mihaela Panaitescu, Laura Enache, Celina Maria Damian, Ramona Marina Grigorescu, Augusta Raluca Gabor, Cristian Andi Nicolae, Cristina Lavinia Nistor, Roxana Trusca

**Affiliations:** 1 ELMAT Laboratory, Faculty of Electrical Engineering, University Politehnica of Bucharest, 313 Splaiul Independentei, 060042 Bucharest, Romania; 2 Polymer Department, National Institute for Research and Development in Chemistry and Petrochemistry, 202 Splaiul Independentei, 060021 Bucharest, Romania; 3 Advanced Polymer Materials Group, University Politehnica of Bucharest, 1–7 Gh. Polizu Street, 011061 Bucharest, Romania; 4 Department of Science and Engineering of Oxide Materials and Nanomaterials, University Politehnica of Bucharest, 1–7 Gh. Polizu Street, 011061 Bucharest, Romania

**Keywords:** dielectric elastomers, mechanical properties, dielectric spectroscopy

## Abstract

The structure–property relationship of dielectric elastomers, as well as the methods of improving the control of this relationship, has been widely studied over the last few years, including in some of our previous works. In this paper, we study the control, improvement, and correlation, for a significant range of temperatures, of the mechanical and dielectric properties of polystyrene-b-(ethylene-co-butylene)-b-styrene (SEBS) and maleic-anhydride-grafted SEBS (SEBS-MA) by using graphite (G) as filler in various concentrations. The aim is to analyze the suitability of these composites for converting electrical energy into mechanical energy or vice versa. The dielectric spectroscopy analysis performed in the frequency range of 10 to 1 MHz and at temperatures between 27 and 77 °C emphasized an exponential increase in real permittivity with G concentration, a low level of dielectric losses (≈10^−3^), as well as the stability of dielectric losses with temperature for high G content. These results correlate well with the increase in mechanical stiffness with an increase in G content for both SEBS/G and SEBS-MA/G composites. The activation energies for the dielectric relaxation processes detected in SEBS/G and SEBS-MA/G composites were also determined and discussed in connection with the mechanical, thermal, and structural properties resulting from thermogravimetric analysis, differential scanning calorimetry, Fourier-transform infrared spectroscopy and X-ray photoelectron spectroscopy analyses.

## 1. Introduction

In recent years, the extremely fast development of high-technology applications in different domains, such as electronics, medicine, or robotics, has resulted in a tremendous demand for new types of very performant transducers. Their development requires very high integration and control of the electric and mechanical performances of their components [1,2,3,4,5,6,7]. To design and develop new transducers tailored for precise applications is quite a challenging task, knowing that the conventional actuating devices used in technical applications have several drawbacks, such as high weight, limited dimensions, restrictive shapes, not enough compactness and flexibility, and sometimes, not high enough energy efficiency [1]. This means that new classes of electroactive materials, i.e., materials able to respond to external electrical stimulation by displaying a significant size or shape change, need to be developed. The research carried out to solve these issues has concluded that electroactive dielectric elastomers have a greater potential than other electroactive materials, such as shape memory alloys, ceramics, conductive polymers, and ionic polymer metal composites [4,5]. This is due to their special properties: a high ratio between power and mass, high electromechanical strain, the ability to respond in a fast way, the ease of their processability, and affordable costs [1,6].

At the same time, due to the constant need for low-power autonomous electronic systems and highly integrated devices, new power sources are required so that the energy existing in the environment can be more efficiently recovered [8]. One possible solution is expected to emerge from research aimed at exploiting the energy-harvesting capabilities of new electroactive materials, these so-called “smart” materials, with already proven sensor and actuator capabilities. Among the new electroactive materials, dielectric elastomers have already been used as part of systems capable of converting the residual energy existing in the environment into usable electrical energy [9,10].

Many studies in the last decade have reported that dielectric elastomers are very promising materials to convert electrical energy into mechanical energy, or vice versa, in several applications, such as electromechanical transducers or energy-harvesting systems [1,9,10,11]. All these studies have had as their final goal to improve the electromechanical sensitivity of the dielectric elastomers through several approaches, such as testing different types and combinations of polymers, with or without adding (nano)particles to make dielectric composites [11]. However, what material property needs to be changed to improve its actuation performance and which figure of merit is best to evaluate the electromechanical conversion abilities of the tested dielectric elastomers are two questions with different answers based on the different approaches reported in the literature [1,4,5,6]. One of the sources of these differences was the mechanism of electrical actuation, which is considered as dominant. Thus, it is well known that when a dielectric elastomer is placed between two electrodes, it electrically actuates through two actuation mechanisms: Maxwell stress, caused by the Coulomb interaction between oppositely charged electrodes, and the true electrostriction effect that originates from the direct coupling between the polarization and the mechanical response [1,2,4]. The first studies emphasizing the remarkable actuation performance of dielectric elastomers, in particular poly[styrene-b-(ethylene-co-butylene)-b-styrene] (SEBS) triblock copolymers, considered that the main actuation mechanism was Maxwell stress, while the electrostrictive mechanism was responsible in general for the actuation behavior of ferroelectric polymers [6]. By considering Maxwell stress as the dominant actuation mechanism, a number of research studies focused on developing dielectric elastomers or elastomer-based composites with high electric permittivity and low stiffness, namely, a low Young modulus [6,9,10,11,12,13]. However, more recent experimental evidence has emphasized that the true electrostriction effect dominates the actuation of the thermoplastic dielectric elastomers, such as SEBS and SEBS-MA, and, moreover, that thermoplastic dielectric elastomers considerably actuate even without a Maxwell stress contribution [1,14].

Therefore, it is a difficult task to differentiate between the numerous dielectric elastomers developed in the last few years and to evaluate which of them is most suitable to act as a transducer between electrical and mechanical energies so as to be used for actuation as a sensor or harvester of mechanical energy. A recent review by Banet et al. [11] aimed to find a method to compare the newly developed dielectric elastomers and to propose a procedure for choosing the most suitable modification to obtain the best elastomer for a certain application. The discussion in that review was focused on the main three parameters that impact the actuation, namely, Young’s modulus (*Y*), the real part of the complex relative permittivity (ε_r_’), and the breakdown field (*E_b_*). Thus, the main goal of that study was to find the best compromise between these parameters, as it is well known that high deformations are obtained in materials with low *Y*, whereas high values of ε_r_’ and *E_b_* determine high electrical energy densities. The main conclusion of that study emphasized that any strategy for improving the electromechanical performances of dielectric elastomers should allow precise control of the dielectric properties and the material stiffness, an increase in both while maintaining a high breakdown field being beneficial for the applications of these materials [11].

In one of our previous works, tough and flexible dielectrics were prepared using graphite (G) as filler in polystyrene-b-(ethylene-co-butylene)-b-polystyrene (SEBS) and maleic-anhydride-grafted SEBS (SEBS-MA) matrices [15]. Simultaneous increases in tensile strain, strength, and Young’s modulus were noticed for SEBS/G and SEBS-MA/G composites compared to unfilled matrices, this remarkable feature being previously reported only for some nanocomposites. Moreover, an exponential variation of the dielectric permittivity with the volume fraction of G was obtained at room temperature.

In this work, we continue the study on SEBS/G and SEBS-MA/G composites to analyze the potential use of these composites in applications for converting electrical energy into mechanical energy or vice versa. Thus, one novelty of the present research is the analysis of the control and correlation of the mechanical and dielectric properties of these composites for the range of temperatures between 27 and 77 °C, whereas the previous tests in [15] had been performed only at room temperature. Hence, we emphasize the temperature effects on the dielectric response in correlation with the dynamic mechanical analysis (DMA) results, while in the previous work, the Young modulus had been determined in tensile tests. Moreover, the analysis of dielectric spectroscopy (DS) data was made this time by using the Havriliak–Negami (HN) dielectric model [16,17] for fitting the experimental spectra obtained at different temperatures. The HN fitting results of the imaginary part of the complex permittivity have emphasized two dielectric relaxations as well as a DC conduction process. The activation energies for the two relaxation processes were determined, and the dielectric spectroscopy results were discussed in connection with the mechanical properties of the SEBS/G and SEBS-MA/G composites, alongside their thermal and structural features determined by thermogravimetric analysis (TGA), differential scanning calorimetry (DSC), Fourier-transform infrared spectroscopy (FTIR), scanning electronic microscopy (SEM), and X-ray photoelectron spectroscopy (XPS).

The range of temperatures for the dielectric spectroscopy tests was chosen to be between 27 and 77 °C because this range covers the usual operating temperatures of electrical and electronic devices, and it is between the glass transition temperatures of the two phases present in SEBS, namely, the poly(ethylene-co-butylene) (PEB) and polystyrene (PS) blocks. The temperature effects on the dielectric behavior of the tested materials have been studied by following both the heating and cooling procedures of the samples.

## 2. Materials and Methods

### 2.1. Materials

Polystyrene-b-(ethylene-co-butylene)-b-styrene (SEBS), with the commercial code Kraton G1652, and maleic-anhydride-grafted polystyrene-b-(ethylene-co-butylene)-b-styrene (SEBS-MA), with the commercial code Kraton FG 1901X, were purchased from Kraton Co. (USA). They have a density close to 0.91 g/cm^3^ and contain 30 wt% styrene. SEBS-MA also contains 1.84 wt% maleic anhydride. The melt flow index at 230 °C with 5 kg is 5.0 g/10 min for SEBS and 17.2 g/10 min for SEBS-MA. Graphite flakes (G), with 90% carbon content and particle sizes between 30 and 50 µm, were purchased from Georg H. Luh. Gmbh (Germany).

### 2.2. Preparation of Samples

Two types of SEBS composites with graphite were obtained in the 50 cm^3^ mixing chamber of a Brabender LabStation at 185 °C with a rotor speed of 60 min^−1^. The melt mixing process of the two components took about 7 min after SEBS or SEBS-MA melting. Immediately after removal from the mixing chamber, the materials were molded for straightening with a two-roll mill, and then, they were compression-molded into plates with a thickness of 0.5 mm using a P200E laboratory press (Dr. Collin, Germany). The materials were heated in the press at 175 °C, with 150 s of preheating and 120 s under 15 MPa, and then rapidly cooled in a cooling cassette. The samples were denoted as SEBS/Gx or SEBS-MA/Gx, with x being the weight concentration of G, which varied between 0 and 20 wt%.

### 2.3. Thermal Characterization

The thermal stability of composites was evaluated by thermogravimetric analyses using a TGA Q5000 (TA Instruments, New Castle, DE, USA). Dynamic runs were carried out from room temperature to 700 °C, with a heating rate of 10°/min, in a nitrogen atmosphere.

DSC analysis was carried out using a DSC Q2000 (TA Instruments, New Castle, DE, USA). Each sample of ~5 mg was placed in an alumina crucible and tested under a helium flow (100 mL/min). The temperature program was an equilibration at 150 °C, followed by a ramp at 10°C/min from −150 to 190 °C, isothermal for 5 min at 190 °C, with a cooling step at 10 °C/min to −150 °C, isothermal for 5 min at −150 °C, and a second heating at the same speed of 10 °C/min up to 190 °C. The melting temperature (*T_m_*) assumed by the PEB block and the values of the glass transition temperature (*T_g_*) of the PEB blocks were taken from both the first and second heating steps.

### 2.4. FTIR Spectroscopy

The FTIR spectra of SEBS and SEBS-MA composites were recorded with a Tensor 37 spectrometer from Bruker Optics (Ettlingen, Germany) in the attenuated total reflectance (ATR) mode. The spectra were collected from 400 to 4000 cm^−1^, with 16 scans and a resolution of 4 cm^−1^.

### 2.5. XPS Analysis

The surface chemical compositions of SEBS and SEBS-MA composites were characterized by X-ray photoelectron spectroscopy using a Thermo Scientific K-Alpha X-ray Photoelectron Spectrometer (XPS) System (Thermo Fisher Scientific, Waltham, MA, USA) with a monochromatic AlKα source (1486.6 eV) at a pressure of 2 × 10^−9^ mbar. The pass energy was 200 eV for the survey spectra and 20 eV for the high-resolution spectra.

### 2.6. SEM Analysis

The morphology of the composites was analyzed by SEM using a Philips/FEI Quanta Inspect microscope (FEI Company, Hillsboro, OR, USA). Before analysis, the samples were fractured in liquid nitrogen and surface-coated with gold. SEM images were recorded at an acceleration voltage of 30 kV.

### 2.7. Dynamic Mechanical Analysis

The storage modulus (G’), loss modulus (G”), and mechanical loss factor (tan δ) were recorded at a constant frequency (1 Hz) as a function of temperature, from room temperature to 150 °C, with a heating rate of 3 °C/min, using a DMA Q800 (TA Instruments, New Castle, DE, USA). The stress was generated in shear mode, which is useful for the examination of low-stiffness materials such as elastomers. The samples, 10 × 10 × 1.3 mm (length × width × thickness), were cut from compression-molded plates and equilibrated at room temperature for 5 min, then heated to 150 °C.

### 2.8. Dielectric Spectroscopy

**Figure 1:**
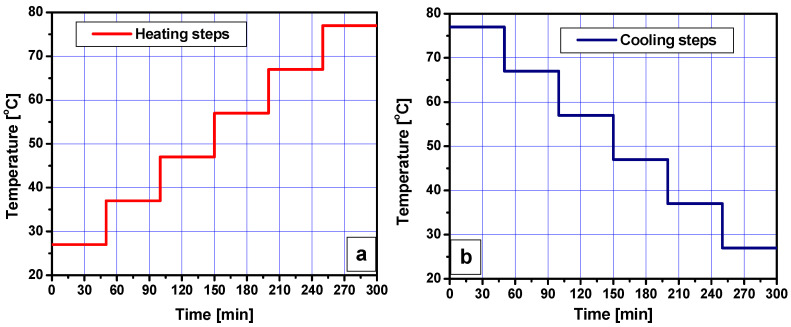
Temperature variation during the dielectric spectroscopy tests: (**a**) heating steps, (**b**) cooling steps.

Broadband dielectric spectroscopy (BDS) measurements were performed using a Novocontrol Alpha-A Analyzer with Active Sample Cell ZGS (Novocontrol Technologies, Montabaur, Germany). The real (ε_r_’) and imaginary (ε_r_’’) parts of the complex relative permittivity were determined over a frequency range from 10^−2^ to 10^6^ Hz, during either a heating procedure or a cooling procedure, between 27 and 77 °C, with steps of 10 °C, as shown in Figure 1. Before measuring a dielectric spectrum, the samples were maintained for 30 min at each temperature under airflow. Two parallel measurements were performed for each sample.

## 3. Results and Discussion

The discussion of the results is focused on the control and improvement of the mechanical and dielectric properties of SEBS by the incorporation of G and by grafting with MA. However, in order to better explain the structure–property relation regarding the mechanical and dielectric properties, as well as the correlation between these properties, we start this section with the presentation of the results, giving first the information on the structure of SEBS, SEBS-MA, and their composites.

### 3.1. Thermal Stability

**Figure 2:**
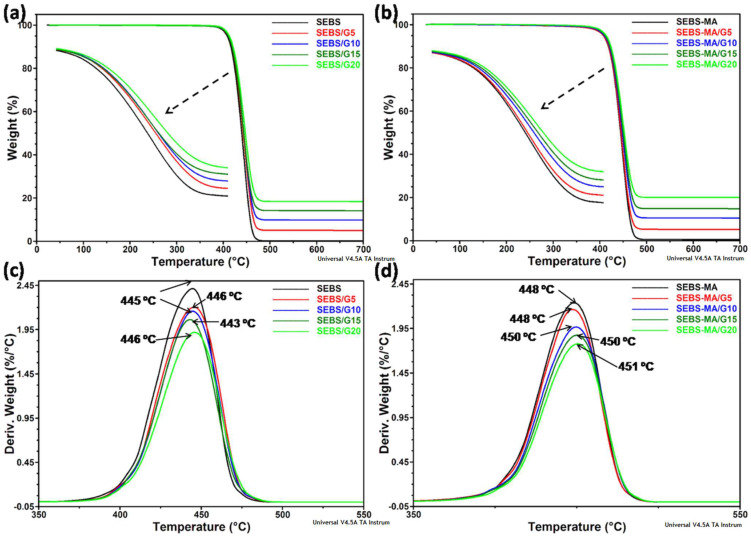
TGA curves of SEBS (**a**) and SEBS-MA composites (**b**); detailed part of the curves shown in the insets; DTG curves of SEBS (**c**) and SEBS-MA (**d**) composites with *T_d_* values noticed on the curves.

Good thermal stability is mandatory for any electrical application, and SEBS copolymers are well known for their high thermal stability and the maintenance of their properties in a wide range of temperatures. Similarly, the influence of G inclusions (0–20 wt%) on the thermal degradation of block copolymer matrices (SEBS and SEBS-MA) is important, and the TGA profiles of block-copolymers and composites are shown in Figure 2.

A single weight loss step was observed for all the samples between 400 and 480 °C, mainly due to the chain scission at the boundary of the PS and PEB phases [18]. It is worth remarking on the high thermal stability of the composites, the weight loss being less than 3%, up to 400 °C, for both the SEBS and SEBS-MA composites. An increase of 1–2 °C in the onset degradation temperature was observed for all the composites compared to the matrices, regardless of the graphite loading.

Moreover, higher degradation temperature was observed for the SEBS-MA composites compared to similar SEBS composites. In particular, an increase in T_50_ (the temperature at 50% weight loss) by 6 °C was obtained for SEBS-MA/G15 compared to the matrix (450 °C instead of 444 °C) and by 4 °C for SEBS/G15 compared to SEBS (444 °C instead of 440 °C). Detailed curves show lower weight loss and increased thermal stability with the increase in the G content (inset of Figure 2a,b). The influence of the matrix type can be better observed by examining the DTG curves: SEBS-MA composites show higher values for T_d_ (the temperature at the maximum weight loss rate) compared to similar SEBS composites (Figure 2c,d). The effect of graphite in improving the thermal stability of polymers was previously signaled [19,20], but the cumulative effect of grafted MA and graphite was not yet reported. Residue values generally reflect the G concentration in the composites.

Remarkably, for all the samples, the weight loss was under 0.1% at 200 °C, a temperature close to the melt processing limit, so no degradation products could occur under this temperature in the absence of oxygen.

### 3.2. DSC Analysis

**Figure 3:**
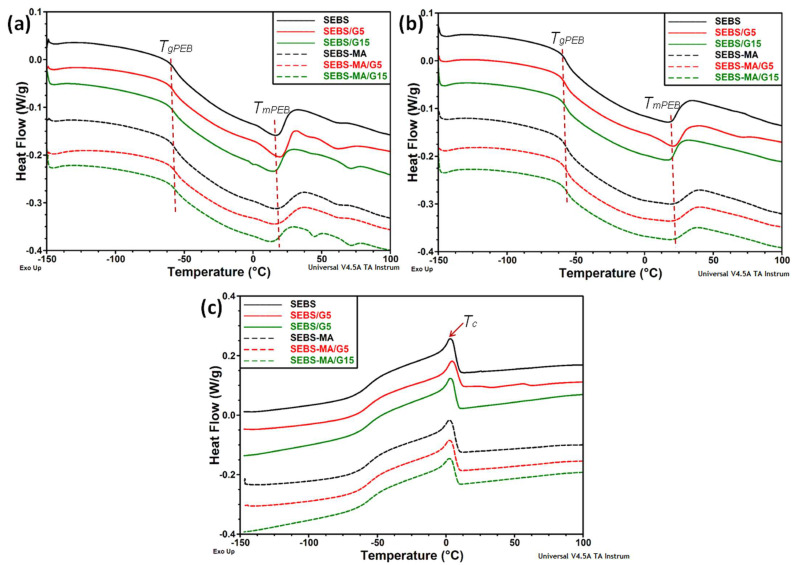
DSC curves of SEBS and SEBS-MA composites: first heating (**a**), second heating (**b**), and cooling (**c**).

Selected samples of unfilled copolymers and composites were characterized by differential scanning calorimetry (DSC) in a wide temperature range to emphasize the glass transition temperatures of both phases, soft PEB and rigid PS. Thus, the DSC curves—first heating, cooling, and second heating—of SEBS, SEBS-MA, and their composites, with 5 and 15 wt% G, are shown in Figure 3a–c. The α-relaxation of PEB middle-blocks (*T_gPEB_*) and the melting of ordered mid-blocks (*T_mPEB_*) can be observed in the first and second heating scans and the crystallization of the PEB blocks in the cooling scan. As a characteristic of SEBS, the glass transition of the PEB blocks is coupled with a broad endothermic peak ascribed to locally ordered PEB domains [21,22]. The glass transition of PEB blocks was observed in all references and composites at around −57 °C, and it was not influenced by the G amount (Table 1).

The endothermic event between −60 and 35 °C in SEBS, SEBS-MA, and their composites can be related to the melting of the ordered part of the PEB blocks (Figure 3). A slight decrease in T_m_ values was observed for SEBS and SEBS-MA composites with a high amount of G compared to their matrices, suggesting a looser structure because of G particles, which probably hindered the order in the PEB middle-blocks. In the case of SEBS composites, the T_m_ values were similar for the first and second heating runs, but they were different for SEBS-MA composites. The increase in T_m_ from 22 and 18 °C (I Heating) to 27 and 25 °C (II Heating) for SEBS-MA/G5 and SEBS-MA/G15, respectively, indicated some interaction at the SEBS-MA/G interface favored by MA groups, knowing that MA is attached to PEB blocks.

The glass transition of PS blocks could not be determined with high accuracy from the DSC diagrams. However, T_gPS_ was around 80 °C for all the samples, and an increase by ≈10 °C in this value was detected in SEBS/G15 compared to the reference SEBS. No detectable difference in T_gPS_ value was observed for the SEBS-MA composites.

Several small calorimetric events were observed between 40 and 80 °C, which were related to the thermal history and the melt processing parameters, and they appeared only in the first heating scan.

### 3.3. FTIR Spectroscopy

**Figure 4:**
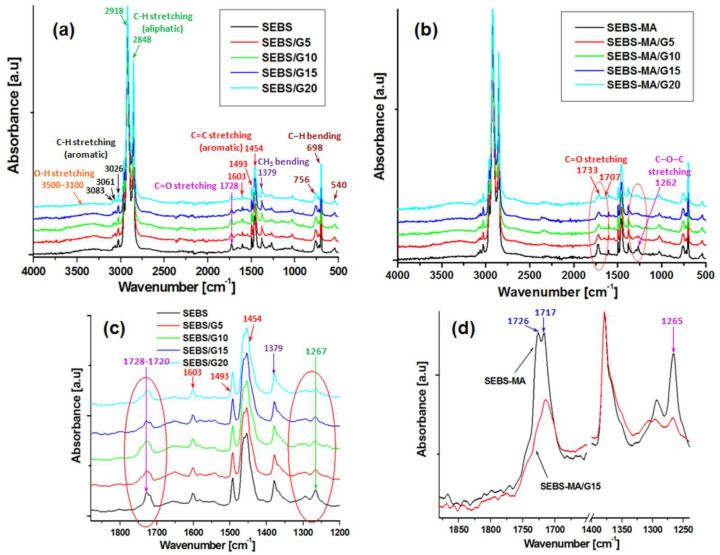
FTIR spectra of SEBS (**a**) and SEBS-MA (**b**) composites; FTIR spectra in the fingerprint region of SEBS (**c**) and SEBS-MA (**d**).

FTIR spectra of the SEBS and SEBS-MA composites are shown in Figure 4a,b. SEBS has the main peaks at 3083/3061/3026 cm^−^^1^ (triple bands—C-H aromatic stretching), 2918/2848 cm^−^^1^ (C-H aliphatic stretching), 1603/1493/1454 cm^−^^1^ (C=C aromatic stretching), 1379 cm^−^^1^ (CH_3_ bending [23]), and 756/698/540 cm^−^^1^ (C−H out-of-plane bending), similar to other observations [24,25]. Moreover, new peaks appear at 1267 and 1728 cm^−^^1^, with a shoulder at 1720 cm^−^^1^ and a hump at 1656 cm^−^^1^, typical for C-O and C=O group stretching [23]. Similarly, a broad absorption rising between 3100 and 3500 cm^−^^1^ due to bonded OH stretching was detected [23]. The presence of carbonyl/carboxyl and hydroxyl groups on the SEBS surface suggests some degradation processes that might have occurred during the melt processing steps (melt compounding and compression molding), which take place at high temperatures (175–185 °C) or during synthesis or prior processing of the original material. All these groups, detected by ATR FTIR on the surface of SEBS, significantly influence dielectric behavior, as discussed in the next section.

Interestingly, the band at 1728–1720 cm^−^^1^ changes the shape in the case of composites by broadening and by the evidence of an obvious shoulder at about 1743 cm^−^^1^, characteristic of the ester bond (Figure 4c, marked with a red circle). This shift from a lower wavenumber to a higher one in the range 1720–1743 cm^−^^1^ shows the transformation of aldehyde or acid groups in ester groups. This is probably due to the reaction of -CHO or -COOH groups from SEBS with some OH groups from the surface of G. In a similar manner, the decrease in the intensity of the peak at 1267 cm^−^^1^ in composites compared to the matrix (Figure 4c, marked with a red circle) supports the involvement of C-O and C=O groups from SEBS in reactions with hydroxyl groups on the G surface [26].

The FTIR spectrum of SEBS-MA shows the bands characteristic to aliphatic and aromatic C-H bonds and aromatic C=C, similar to SEBS. In SEBS-MA, maleic anhydride can be in a ring form or in a hydrolyzed form, showing dicarboxylic acid groups; the ring form shows the peaks characteristic to the vibrational coupling of the two C=O groups at 1856, 1780, and 1720 cm^−^^1^ [27,28] and maleic acid shows signals at 1707 cm^−^^1^, characteristic to the intramolecular H-bonded C=O stretch, at 1733 cm^−^^1^, characteristic to C=O stretch, and at 1262 cm^−^^1^, due to C–O–C stretching vibrations [29].

One can observe from Figure 4d that no important peak appears between 1870 and 1750 cm^−^^1^, and only a double peak is observed at 1726/1717 cm^−^^1^. A medium-intensity peak is observed at 1265 cm^−^^1^. Therefore, it can be concluded that the MA ring in SEBS-MA was most frequently in the open state, with carboxylic groups. Interestingly, the band at 1726–1717 cm^−^^1^ changed significantly in both shape and intensity in the case of composites, regardless of the G concentration: it became broader, and its intensity was smaller. This means that carbonyl or carboxyl groups were consumed, and probably an esterification reaction occurred between the (hydrolyzed) maleic anhydride groups (-CHO or -COOH) from SEBS-MA and the hydroxyl groups on the surface of G [26,30,31]. As expected, the peak at 1265 cm^−^^1^ showed the same behavior.

### 3.4. XPS Analysis

**Figure 5:**
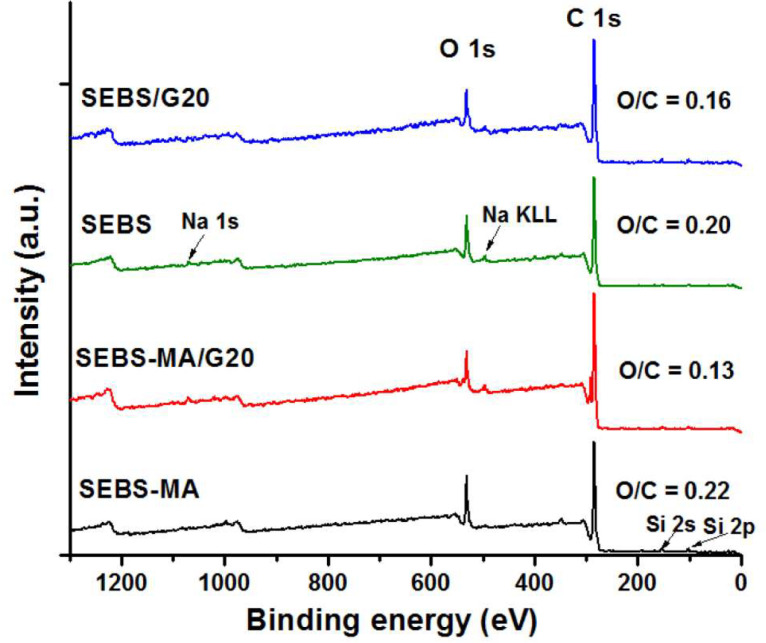
XPS survey spectra of SEBS and SEBS-MA and their composites with 20 wt% G.

**Figure 6:**
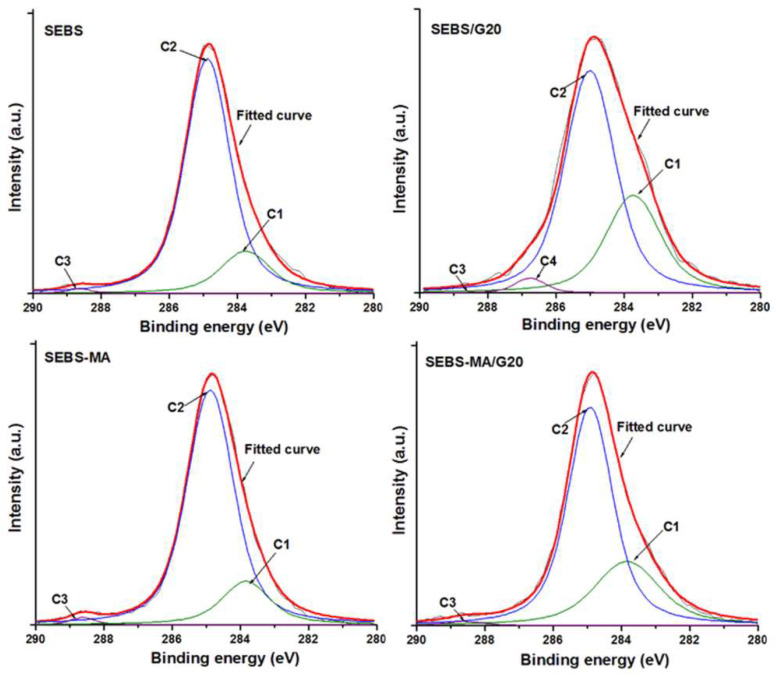
XPS high-resolution spectra of SEBS and SEBS-MA and their composites with 20 wt% G.

XPS analysis provides information about the elemental composition and chemical state on the surface of the samples, which is also very useful for dielectric spectroscopy analysis. An XPS survey and the high-resolution spectra of SEBS and SEBS-MA and their composites with the highest amounts of G are shown in Figure 5 and Figure 6 and Table 2. The XPS survey spectra indicated that C and O are the predominant elements in all these samples: the peak at 285 eV corresponds to the binding energy (BE) of C1s and that at 532 eV to the BE of O1s.

The O element was expected to appear in small amounts in SEBS-MA and its composites due to the MA groups. The presence of O on the surface of all the samples, references, and composites may have resulted from the oxidation of SEBS during the melt processing process and aging during storage in laboratory conditions (RH 50% and 23 °C) for more than a month before characterization. These observations are in line with the FTIR results, which emphasized the presence of carbonyl or carboxyl and hydroxyl groups on the SEBS surface.

However, the surface O/C atomic ratio was different in the composites compared to SEBS and SEBS-MA, respectively (Figure 5). The lower amount of O in the composites may have been caused by the incorporation of G and its influence on the oxidation of the samples. Indeed, a study on styrene butadiene rubber (SBR) modified by graphene has shown that O/C atomic ratios of 0.11 and 0.22 were obtained for SBR before and after aging, respectively, but lower values were noticed after the incorporation of 7 phr of graphene: 0.08 and 0.14 (before and after aging) [32]. Moreover, the slightly lower O/C value obtained for SEBS-MA/G20 compared to SEBS/G20 could be a result of the weaker interface in the case of SEBS/G20, which led to a worse dispersion of G and the accumulation of the polymer matrix at the surface.

Another important feature is the presence of supplementary peaks belonging to Si 2p—Si 2s (103 and 152 eV) and Na 1s (1071 eV) in the survey spectra of both references and composites (Figure 5). This suggests the presence of a filler (such as a silicate) in the original SEBS and SEBS-MA, which are commercial block copolymers. Indeed, a previous work signaled the presence of talc in a similar SEBS block copolymer [22]. The presence of a silicate in the references could also explain their higher proportion of the O element.

High-resolution C 1s spectra of block copolymers and composites show two main peaks at about 284 and 285 eV, which are assigned to the chemical state of carbon sp2 (C=C) and sp3 (C-C/C-H), respectively, and a bump at 288.6 eV, characteristic to O-C-O or C=O. Besides these, a small peak at 286.8 was visible in SEBS/G20 and was ascribed to C-O. The different bonds between C and O, resulting from the oxidation of SEBS and SEBS-MA, are in small amounts (below 3 at%) on the surface of the samples, but they can account for the accumulation of charges and the relaxation observed in the dielectric spectra, which are discussed further.

### 3.5. Dynamic Mechanical Analysis

**Figure 7:**
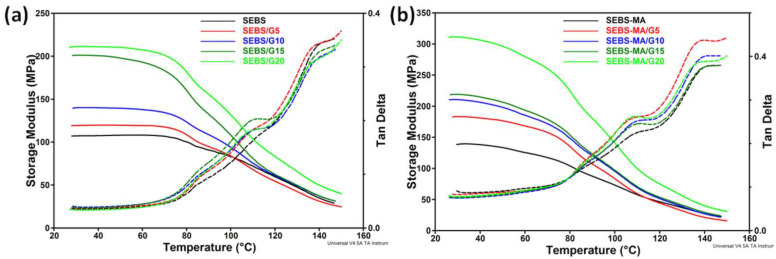
DMA storage modulus and tan δ curves for SEBS (**a**) and SEBS-MA (**b**) composites.

The storage modulus (G’) and the mechanical tan δ variations with temperature are shown in Figure 7. A double broad peak (~85 °C; ~110 °C) was observed in the tan δ curve for the SEBS composites (Figure 7a) with a higher amount of G, and only a weak peak at about 85 °C for SEBS and SEBS/G5. A very weak deviation was observed close to 110 °C for SEBS/G5. Considering the DSC results and previous reports [33], the double peak could be ascribed to the glass transition of the PS blocks, the lower temperature shoulder to the “unrestricted” PS blocks, and the higher temperature peak to the PS chains close to the G surface.

It is worth remarking on the important increase in storage modulus with the increase in G content in SEBS or SEBS-MA composites. The storage modulus of SEBS-MA/G20 was higher by 125% compared to the SEBS-MA matrix and that of SEBS/G20, with 97% vs. the SEBS matrix. This shows both the reinforcing effect of graphite and the compatibilizing effect of MA, which increased the strength at the interface and reduced intermolecular slippage [34], leading to a higher increase in storage modulus.

DMA results for 1 Hz and increasing temperature reveal the increase in mechanical stiffness with G content for both SEBS/G and SEBS-MA/G composites. This increase is important in the glassy region of PS blocks, especially between 30 and 80 °C, showing that the G presence leads to the stiffening of the polymeric chains and to the reinforcing of the SEBS structure. Even though the reinforcing mechanism is not completely understood, the immobilization of polymer chains around particles and the creation of flexible nets of polymer chains between particles are the most agreed-upon mechanisms [33]. Hence, the modification of the molecular dynamics close to the polymer–particle interface is considered to be the main cause of the increase in the storage modulus [35]. Thus, in the case of the SEBS/G composite, we suppose that the stiffening was mainly determined by the fixation of PS blocks on G particles by π-π interactions between the benzene quadrupoles of graphite/graphene flakes and those of PS blocks. Kocman et al. showed that the quadrupolar electric field that the carbon atoms exhibit in aromatic compounds due to electron distribution is high enough to contribute significantly to intermolecular interaction, either in the case of small flat structures, such as graphene flakes with sizes smaller than 100 Å, or when considerably large graphene flakes of sizes of some μm or more are corrugated or bent [36]. When the graphene sheets are flat, the quadrupolar field near the surface vanishes with increasing sheet size. In our case, the SEBS/G and SEBS-MA/G composites contain graphene flakes of some tens of μm, which are bent or corrugated, as shown by SEM analysis in our previous paper [15]. Thus, high quadrupolar intermolecular interactions are expected between graphene and graphite bends and folds and the rigid PS blocks of SEBS, with a strong impact on the dielectric behavior of the SEBS/G and SEBS-MA/G composites, as discussed in the next section. Most of these interactions are thought to be attractive since the most favorable π stacking geometries are the parallel, edge-to-face, or T-shaped displacements [37].

The storage modulus was higher for SEBS-MA than for SEBS, which shows that the presence of MA grafts enhances the polymer chain immobilization. Moreover, the storage modulus exhibited a higher increase in the function of G content for SEBS-MA/G composites than for SEBS/G composites. This can be due to the interactions between the G and MA groups of SEBS-MA through hydrogen bonds between MA and surface carbonyl/carboxyl groups on G, which, besides the quadripolar interactions between G and SEBS, influence the molecular dynamics at the filler–polymer interface and, consequently, the reinforcing strength.

Moreover, the storage modulus variation with temperature is different for SEBS-MA and its composites compared to that for SEBS and its composites. While the storage modulus is quite constant for SEBS and SEBS/G composites up to 70 °C, it continuously decreases with temperature for SEBS-MA and SEBS-MA/G composites, suggesting a lower onset and/or a higher range of the phase transition of styrene blocks for the latter. This decrease in storage modulus at temperatures lower than 70 °C for SEBS-MA and SEBS-MA/G composites is probably due to their lower average molecular weight (*M_n_*) and higher melt flow index (MFI) compared to the ones for SEBS and SEBS/G composites. Indeed, the MFI measured in the same conditions is 5.0 g/10 min for SEBS and 17.2 g/10 min for SEBS-MA, while *M_n_* is 79,100 for SEBS and 58,000 for SEBS-MA, according to the producer data.

Moreover, from the DSC results in Figure 3, it can be seen that the *T_g_* of the PEB regions was not affected by the G content; therefore, the G presence did not influence the molecular dynamics in PEB blocks. This confirms that G particles are connected with SEBS mainly through π-π interactions between G and PS blocks.

### 3.6. SEM Analysis of Composites

**Figure 8:**
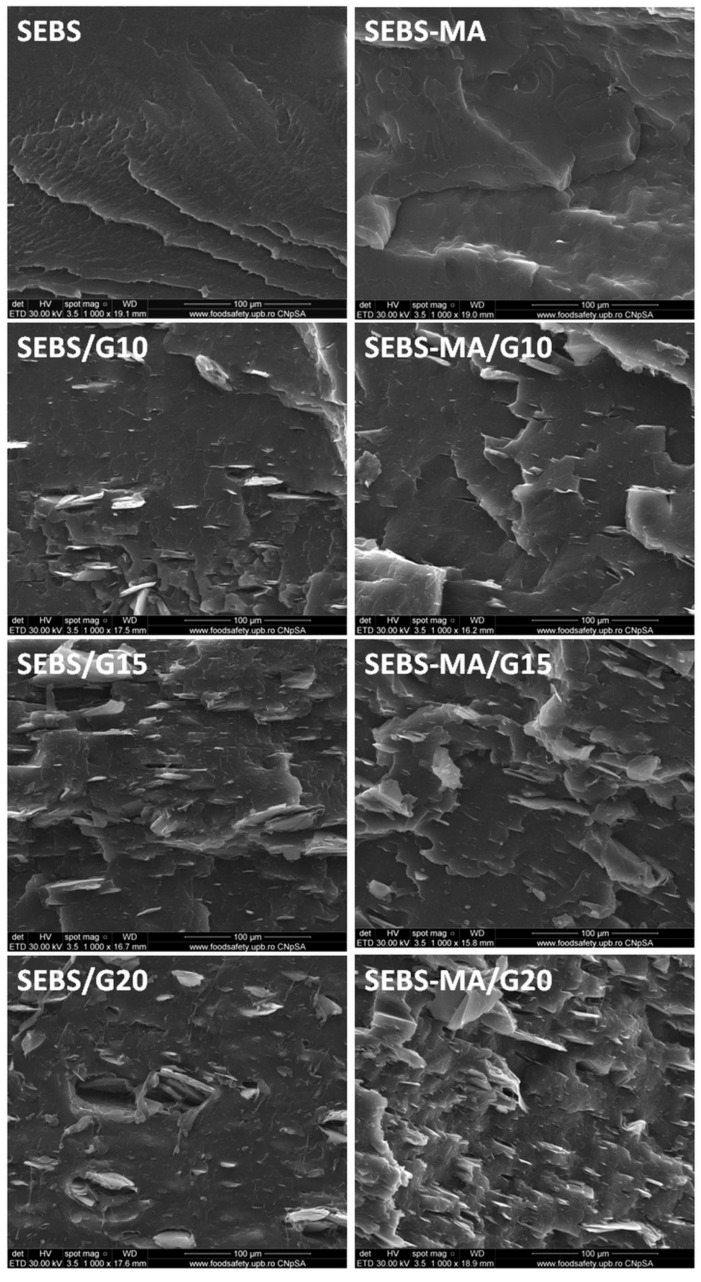
SEM images (×1000) of SEBS, SEBS-MA, and their composites.

The dispersion of the G filler in SEBS and SEBS-MA composites was analyzed by SEM (Figure 8) to better understand the influence of the filler on the properties of composites. SEBS and SEBS-MA have different aspects in the fracture, a better-organized structure being observed in the case of SEBS. The G filler appears in SEM images in the form of stacks of graphene layers characterized by micron widths and nanometric thicknesses. Well-dispersed multilayer graphene stacks were observed in both matrices at 10 and 15 wt% concentrations. However, better dispersion of G and thinner stacks was detected in the fracture of SEBS-MA/G composites. The differences between SEBS/G and SEBS-MA/G composites were obvious at higher concentrations of G (Figure 8): well-dispersed graphene stacks were observed in the fracture of SEBS-MA/20G and clusters, along with holes in the fracture of SEBS/20G. This points out once more the great influence of the MA grafts on the strength of the polymer–filler interface.

**Figure 9:**
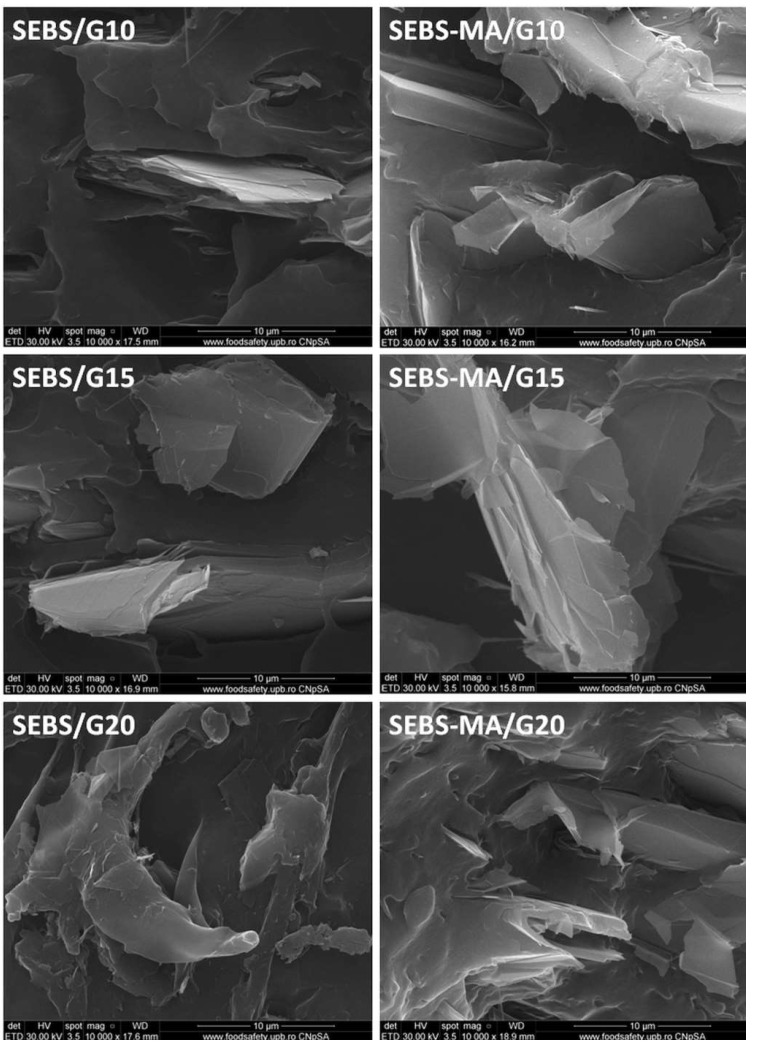
SEM images (×10,000) of SEBS composites and SEBS-MA composites.

The exfoliation of G, which probably occurred during melt compounding, is more clearly noticed in the higher magnification SEM images (Figure 9). Many sheets detached from the stacks, along with thicker stacks, may be observed in the SEM images of all composites. The thickness of the sheets was in the range of 10–50 nm in the SEBS-MA/G composites and over 70 nm in the SEBS/G composites, showing that more than 30 graphene layers were stacked together [15] in these detached sheets and many more layers in stacks. This is probably the influence of the melt processing of the composites. The exfoliation of graphite in a molten polymer under the influence of high-shear stresses formed during melt compounding has already been emphasized for several polymers [15,38].

### 3.7. Dielectric Spectroscopy Analysis

The dielectric spectra of real and imaginary parts of the complex relative permittivity (ε_r_*’* and ε_r_*’’*) and the dielectric loss tangent (tan δ) are shown and analyzed in this section.

**Figure 10:**
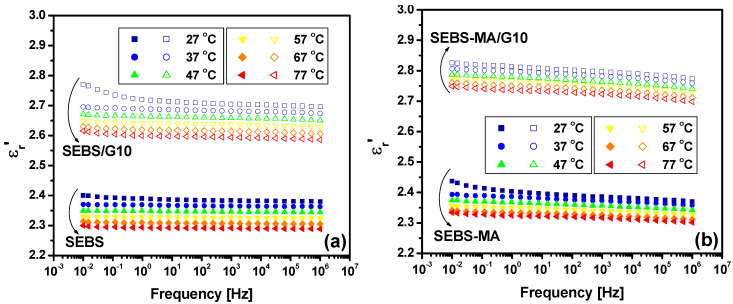
Dielectric spectra of ε_r_’ for SEBS vs. SEBS/G10 composites (**a**) and for SEBS-MA vs. SEBS-MA/G10 composites (**b**) during the heating procedure.

**Figure 11:**
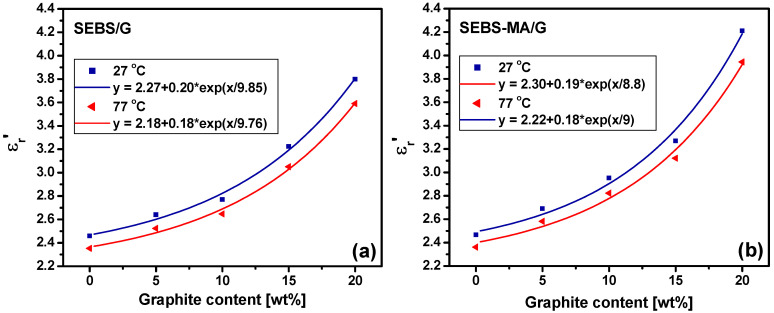
Variation of ε_r_’ with G concentration for SEBS/G (**a**) and SEBS-MA/G (**b**) composites during the cooling procedure at 1 Hz (experimental data—symbols; exponential fit—lines).

This discussion starts with the analysis of the results in Figure 10 and Figure 11 on the frequency variation of ε_r_*’* at different temperatures between 27 and 77 °C during the heating and cooling procedures applied to the tested samples.

The real permittivity ε_r_*’* has quite a small variation with frequency in the studied range for both SEBS/G and SEBS-MA/G composites as well as for the neat polymers. This shows that the dielectric activity is not highly affected by the electric field frequency in this range, as can be seen in Figure 10. This is because, in this frequency range, the polarization is due, on one hand, to the segmental movement from the completely amorphous PEB blocks with highly mobile chains at the tested temperatures that are higher than both T_gPEB_ and T_mPEB_ and, on the other hand, to the orientation of the lateral groups, most of them resulting from oxidation (OH, aldehydes, carboxylic acids or esters), which is present throughout the entire frequency range. The relaxation of the lateral groups is expected to occur only at frequencies over 10^6^ Hz, which is outside the frequency range studied here. The ε_r_*’* values show a small decrease with temperature (Figure 10) because of thermal agitation, which opposes the dipole orientation.

An increase in the ε_r_*’* values with G concentration is noticed in both SEBS/G composites (Figure 10a) and SEBS-MA/G composites (Figure 10b) over the entire range of frequencies. The increase in the ε_r_*’* values with G content is seen at all the tested temperatures, no matter what the sense of reaching that temperature was, by heating (Figure 10) or cooling (Figure 11) the samples.

Moreover, the increase in ε_r_*’* with G concentration follows an exponential law for both SEBS/G and SEBS-MA/G composites, as can be seen in the plots of ε_r_*’* vs. G level at 1 Hz (Figure 11). This increase in ε_r_*’* values with filler content for all frequencies is in line with a similar behavior previously remarked on for these composites but only at room temperature [15]. This exponential increase in ε_r_*’* with G content has almost the same rate at 27 °C as at 77 °C for either SEBS or SEBS-MA composites, showing that the same relaxation mechanisms are present in these composites no matter the temperature in this range. A possible explanation of the increase in ε_r_*’* with G content can be that the G presence inside SEBS and SEBS-MA polymers leads to the formation in PEB blocks of new side groups able to determine easier and more numerous β-relaxations than in neat polymers. These new lateral groups can be esters resulting in composites from the transformation of several carboxylic acids existing in the unfilled polymers, as noticed from the FTIR result analysis. The presence of esters in composites instead of the acids from the neat polymer (either SEBS or SEBS-MA) means that strong intermolecular forces due to hydrogen bonds, present in the case of acids, are replaced by weaker dipole–dipole interactions in the case of esters [39]. Thus, the local electric field may orient the lateral ester dipoles in composites more easily than the acid dipoles in neat polymers, explaining the increase in ε_r_*’* with G in the case of composites. Moreover, even the local electric field acting in composites can be higher than in neat polymers due to the high quadrupolar intermolecular interactions between graphene and graphite bends and folds and the rigid PS blocks of SEBS, as discussed in the previous section. Furthermore, the increase in ε_r_*’* in composites is also the result of a higher dipole moment of esters compared with the carboxylic acids from the polymer without G. Indeed, -COOCH3 has a higher dipole moment than –COOH in both aliphatic compounds (6 × 10^−^^30^ Cm vs. 5.7 × 10^−^^30^ Cm) and aromatic compounds (6 × 10^−^^30^ Cm vs. 5.3 × 10^−^^30^ Cm) [40].

The exponential increase in ε_r_’ values with G level can also be due to the cleavage of G flakes due to the shearing forces at the SEBS-G or SEBS/MA-G interface [15]. The surface of these G fragments, resulting from cleavage, interacts with oxygen, leading to new groups, especially esters, and, hence, new lateral dipoles that contribute to the higher dielectric activity in composites. The ε_r_’ variation with G in Figure 9 is slightly more rapid in the case of SEBS-MA composites compared to SEBS ones, suggesting a slightly higher number of lateral dipoles due to side groups in the SEBS-MA composites, resulting either from the opening of MA rings or from a more intense fragmentation of G flakes in the case of SEBS-MA composites [15]. This small increase in real permittivity in SEBS-MA composites compared to SEBS composites shows that the contribution of MA grafts to the new lateral dipoles is only marginal with respect to that of G flakes.

The exponential increase in ε_r_’ with graphite content is very important for the technical applications of these composites, emphasizing the possibility of controlling the dielectric behavior and precisely tuning the permittivity and, hence, the electrical energy density by graphite concentration in composites.

This result is a small but important step towards tailored dielectric elastomers composites for sensing, actuation, or energy-harvesting applications. Certainly, the control of the permittivity values with the filler content should be continuously improved so as to take place at a significantly higher rate and up to much higher values of ε_r_’.

**Figure 12:**
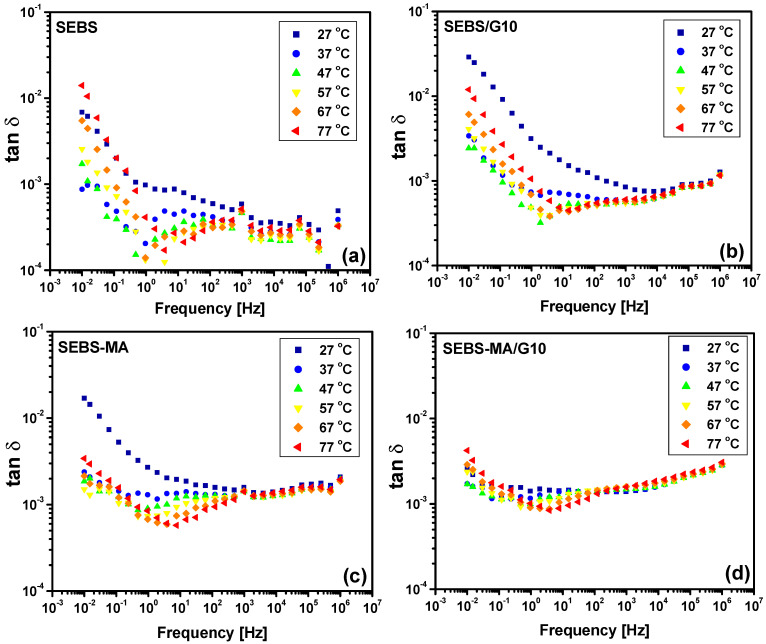
Dielectric spectra of tan δ for SEBS (**a**), SEBS/G10 composites (**b**), SEBS-MA (**c**), and SEBS-MA/G10 composites (**d**) during the heating procedure.

**Figure 13:**
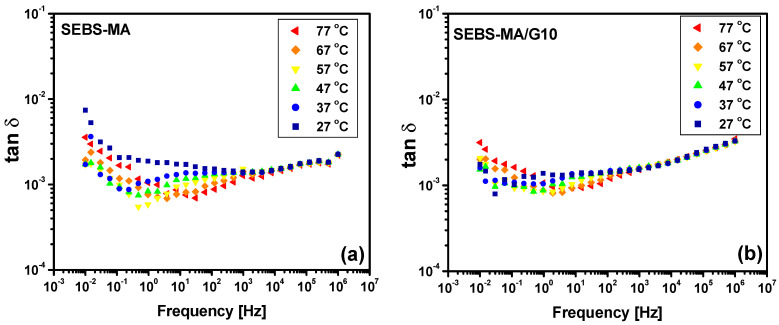
Dielectric spectra of tan δ for SEBS-MA (**a**) and SEBS-MA/G10 composites (**b**) during the cooling procedure.

**Figure 14:**
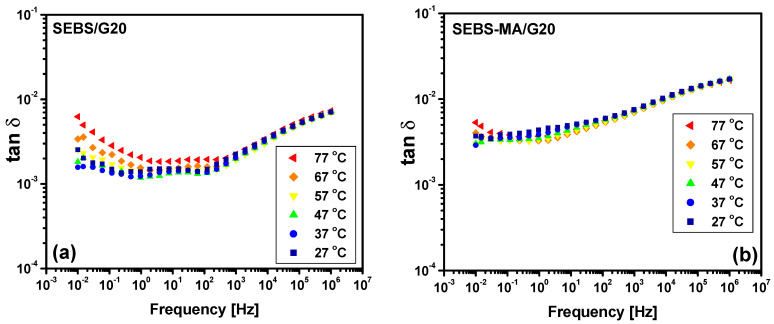
Dielectric spectra of tan δ for SEBS/G20 composites (**a**) and SEBS-MA/G20 composites (**b**) during the cooling procedure.

The polymeric chains are more stiff in composites due to the G presence, especially in SEBS-MA/G composites, as seen from DMA analysis, so the movements of quasi-mobile charges and/or dipoles are less affected by the temperature variation. The results from Figure 12 and Figure 13 confirm this, showing that the temperature influence on the dielectric loss tangent (tan δ) variation of the SEBS-MA/G10 composite is less important than in the case of the neat polymer or in the case of the SEBS-G10 composite. This improved dielectric stability of tan δ with respect to the temperature variation increases with the G content, as can be seen from the results in Figure 14. Indeed, the tan δ spectra for the SEBS-MA/G20 composite at all the studied temperatures (Figure 14b) are closer to each other than in the case of the SEBS-MA/G10 composite (Figure 13b). The results in Figure 12 and Figure 13 show a very small difference between the results in the heating procedure with respect to those from the cooling procedure, and this is due to the thermal inertia of the materials.

The more stable behavior of the dielectric losses with temperature, seen in the composites, is also supported by the XPS results, which show that the number of sp2 carbon atoms increases considerably with G in composites; they practically double at 20% G concentration compared to neat polymers for both SEBS/G composites (32.1 compared to 17) and SEBS-MA/G composites (29.9 compared to 16.6). On the other hand, the number of sp3 carbon atoms decreases significantly with G content with respect to the unfilled polymer for both SEBS/G20 (64.9 compared to 82.1) and SEBS-MA/G20 (69 compared to 81.9). These results suggest that the number of carbon single bonds decreases, whereas the number of carbon double bonds increases, in composites with respect to the unfilled polymer, leading to an increased number of π bonds and, hence, to strong π-π interactions between PS and G, which determines the high stability of dielectric losses with temperature variation.

The high stability of the dielectric loss tangent at temperature variations in the range between 27 and 77 °C, which increases the function of the G content, especially in SEBS-MA/G composites, shows, once again, how the dielectric properties can be controlled by using G as filler at different concentrations.

Besides the above discussion on the stability of dielectric losses with temperature variation, seen in composites with high G concentrations, the low level of dielectric losses for both SEBS/G and SEBS-MA/G composites as well as for the unfilled SEBS and SEBS-MA polymers should be noted. The tan δ values are in the range of 10^−^^4^–10^−^^2^, with a level of 10^−^^3^ for all the tested composites up to hundreds of kHz, which are the usual frequencies for energy-harvesting systems.

To analyze quantitatively the processes revealed by the dielectric spectra obtained for the SEBS and SEBS/MA composites, one should first identify the dielectric relaxations present in the spectra. As is well known, two dielectric relaxations (labeled α and β) can usually be found in the dielectric spectra of polymers in the frequency range of 10^−^^2^–10^6^ Hz above room temperature. The α-relaxation is correlated with the main-chain movement, corresponding to a transition from the glassy state to the rubbery state, as the temperature rises above *T_g_*, whereas the β-relaxation is related to the local motions, such as the rotation of side groups. Furthermore, as the dielectric materials tested in this study are not homogeneous, different interfaces being present either between the PEB and PS blocks in SEBS and SEBS/MA or between the G particles and copolymer matrices, another relaxation can be present in the dielectric spectrum, namely, the interfacial or Maxwell–Wagner–Sillars (MWS) relaxation, due to the charge trapping at these interfaces. Moreover, the free carriers present in copolymers and their composites are responsible for a conduction current that is usually emphasized by the increase in dielectric losses toward lower frequencies.

Thus, taking into account the above considerations, one can identify in the frequency variation of the dielectric loss tangent (tan δ), shown in Figure 12, Figure 13 and Figure 14, two main peaks for both neat polymers SEBS and SEBS-MA as well as for their composites. One peak is located in the medium frequency (MF) range (10–10^2^ Hz), and the other one is in the high frequency (HF) range, around 10^5^–10^6^ Hz. Besides these peaks, there is an increase in the values of tan δ at low frequencies, this being due to a combined effect of DC conduction and electrode polarization. It is interesting to note that this behavior at low frequencies is due to the translation movement of mobile charges, which diminishes significantly with the content of G in SEBS-MA/G composites, suggesting a reduction of the number and mobility of mobile charges in these composites.

The MF peak emphasizes an MWS interfacial polarization, also remarked on in other recent studies on the dielectric behavior of SEBS [17,41]. Ions (possibly oxygen) coming from impurities can accumulate at both the S-EB interface and the interface between G and different SEBS blocks. Supplementary charge accumulations can develop in SEBS-MA/G composites at the interface between G and PEB blocks, where there are hydrogen bonds through opened MA rings. Some air can enter together with the introduction of G powder in the melt polymer during the composite fabrication, thus explaining the oxygen molecules found inside composites by FTIR and XPS [17,41].

The HF peak seen in composites indicates a β-relaxation of oxygen-containing groups from the G surface, especially carboxylate esters produced by the transformation of the carboxylic acids from SEBS and SEBS-MA following G introduction, but also acids and aldehydes that are not transformed in esters. These dipoles can be responsible for the β-relaxation also seen in neat polymers, but here with a considerably higher number of acids and aldehydes than esters, which are expected to be only a few.

Once the dielectric activity in the tested materials was identified, we then continued with the modeling of the spectra. Since dielectric relaxations and the effect of conduction may commonly overlap in a dielectric spectrum, parametric functions are usually employed to separate them and facilitate the analysis of their temperature and frequency dependence. One of the most utilized functions to model a broad and asymmetric distribution of relaxation times is the Havriliak–Negami (HN) function; in contrast, a power-low function is usually used for a conductivity term. Considering all the above, we decided to analyze the non-Debye relaxations from our spectra and to fit the experimental data with the HN model [16]. Thus, we used Equation (1), where the first term accounts for the contribution of the charge carriers on the molecular dynamics and the others are HN terms accounting for the two relaxations remarked on in the dielectric loss spectra obtained for our materials.

(1) $${\underline{\varepsilon }}_{r}\left(\omega \right)=\varepsilon_{r}^{’}\left(\omega \right)-j\varepsilon_{r}^{\prime \prime }\left(\omega \right)=-j{\left(\frac{\sigma_{\mathrm{DC}}}{\varepsilon_{0}\omega }\right)}^{N}+\underset{k}{\sum }\left[\frac{{\Delta \varepsilon }_{k}}{{\left(1+{\left(j{\omega \tau }_{\mathrm{HN}}\right)}^{\alpha }\right)}^{\beta }}+\varepsilon_{\infty }\right]$$

In Equation (1), σ_DC_ is DC conductivity, the exponent N is an indicator of the nature of the electrical conduction process of charge carriers, ε_r_*’*(ω) and ε_r_*’’*(ω) are the real and imaginary parts of the complex permittivity ε_r_, ω = 2πf is the angular frequency, ε_0_ is the vacuum permittivity, *k* equals the number of relaxations of the dielectric response, Δε_k_ is the relaxation strength, τ_HN_ is the HN relaxation time, and α and β are parameters that quantify the width and asymmetry of a given relaxation peak of the loss factor ε_r_*’’*(ω).

**Figure 15:**
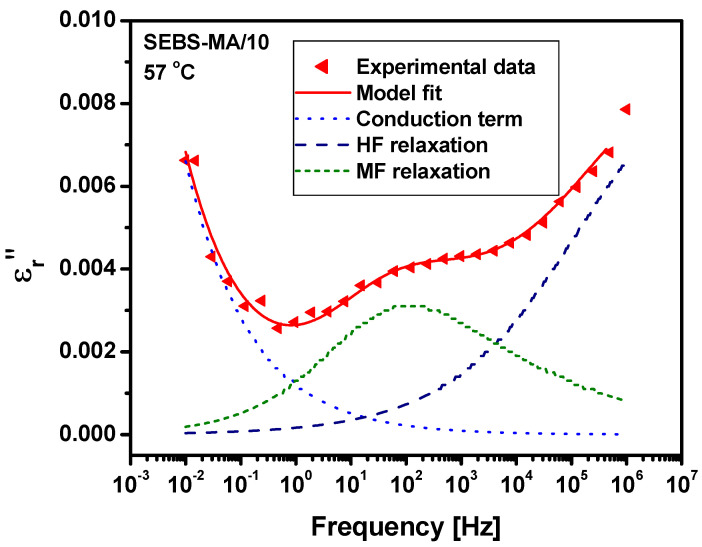
The dielectric spectrum of imaginary permittivity ε_r_’’ of the SEBS-MA/G10 composite at 57 °C, modeled according to Equation (1).

An example of the use of Equation (1) to fit the experimental data is presented in Figure 15, which shows the frequency variation of the imaginary permittivity ε_r_*’’* for the SEBS-MA/G10 composite at 57 °C.

The actual relaxation time τ_max_ was calculated as in Equation (2) [17], and then the frequency f_max_ where the relaxation occurs was determined by *f_max_* = 1/(2πτ_max_).

(2) $$\tau_{\max}=\tau_{\mathrm{HN}}{\left[\sin\left(\frac{\pi \alpha \beta }{2\left(\beta +1\right)}\right)/\sin\left(\frac{\pi \alpha }{2\left(\beta +1\right)}\right)\right]}^{1/\alpha }{}_{}\;$$

**Figure 16:**
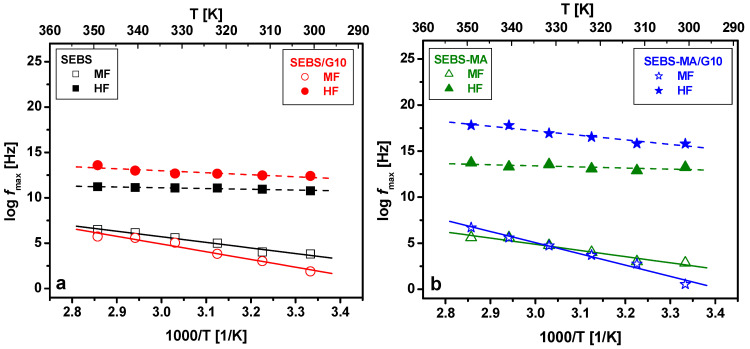
Arrhenius plots showing the variation with temperature of MF and HF peak frequencies *f*_max_ corresponding to MWS and β relaxations in: SEBS/G composites (**a**) and SEBS-MA/G composites (**b**).

Arrhenius plots obtained by processing the dielectric spectra obtained during the heating procedure between 27 and 77 °C are presented in Figure 16. These plots show the variation with temperature of MF and HF peak frequencies corresponding to MWS and β relaxations in SEBS/G and SEBS-MA/G composites.

The activation energy *w_a_* (Table 3) corresponding to MWS relaxation has very close values in SEBS and SEBS-MA neat polymers, showing that the MA presence does not significantly affect the number of mobile charges nor the charge dynamics near the S-EB interface.

As for the composites, the MWS activation energy increases with G content for both SEBS- and SEBS-MA-based composites, indicating reduced charge mobility due to the fixation of S blocks on G as well as a slowdown of the charge movement at the S–EB interface because of the electric field generated by the π-π quadrupolar interactions between the G and S rings. This field can also be the cause of a possible charge accumulation at the G-S interface.

The MWS activation energy is higher for SEBS-MA/G10 (1.05 eV) compared to SEBS/G10 (0.73 eV), which can be explained by the reduced mobility of the quasi-mobile charges due to the supplementary stiffening of the SEBS chains by hydrogen bonds between G and PEB through MA groups. It should be remarked on that the effect of MWS relaxation on the ε_r_*’* values is insignificant in both neat polymers and composites, showing that the local electric field, due to the charges accumulated on the interfaces, is considerably lower than the external field, due to the voltage applied to the electrodes.

The frequency of maximal loss (*f_max_*) corresponding to the HF peak is higher in SEBS/G and SEBS-MA/G composites than in the unfilled polymer at all tested temperatures, as seen in Figure 16. Furthermore, *f_max_* is higher for SEBS-MA and its composites than in the case of SEBS and SEBS/G composites, showing that the β-relaxation of the side groups (carboxylic acids, esters, aldehydes) arises at higher frequencies, which means that the dipoles are able to follow faster electric field oscillations. The orientation of these dipoles is facilitated in the studied temperature range by an enhanced segmental movement of S blocks in SEBS-MA and SEBS-MA/G composites due to a lower molecular weight than in the case of SEBS and its composites. This segmental movement is also emphasized by DMA analysis, where an increase in S blocks flexibility is indicated by the decrease in storage modulus at temperatures lower than 70 °C for SEBS-MA and SEBS-MA/G composites.

However, even if the presence of MA grafts leads to more flexible S regions, determining the shifting of the HF peak towards higher frequencies, the orientation of the side dipoles requires more energy from the electric field in these materials compared to those without MA. Thus, the activation energy for the β-relaxation is higher in MA-grafted SEBS composites, e.g., 0.42 eV for SEBS-MA/G10 compared with 0.19 eV for SEBS/G10.

The analysis of the dielectric spectroscopy results shows that the temperature dependence of the relaxation frequency obeys the Arrhenius equation, i.e., the plot of log (*f_max_*) versus *T*^−1^ gives a straight line (Figure 16) for both the MWS relaxation (MF peak) and the β-relaxation (HF peak). This means that in the analyzed temperature range, none of the two ε_r_*’’* peaks found in the dielectric spectra correspond to molecular processes associated with the glass transition temperature *T_g_* as it is known that the temperature dependence of the relaxation frequency of the molecular process associated with *T_g_* does not conform to Arrhenius law, the plot log (*f_max_*) versus *T*^−1^ being curved as if the activation energy were increasing towards lower temperatures [40]. Indeed, none of the SEBS blocks have phase transitions in the studied temperature range, which is much higher than the *T_g_* of the PEB blocks (≈−57 °C, as shown by our DSC results) but lower than the *T_g_* of the PS blocks (90–100 °C, as reported in other studies [17]). Thus, in the temperature range where we made the dielectric tests, the PEB blocks were very flexible because even the melting temperature *T_mPEB_* was exceeded, which implies a strong segmental movement of PEB chains over the whole frequency range and, hence, the absence of any α-relaxation peak in our dielectric spectra.

The results presented in this paper show that the cumulative effect of grafted MA and G filler on the increase in mechanical stiffness, the exponential increase in real permittivity with filler content, and the increase in the stability of the dielectric loss tangent at the temperature variation of SEBS-MA/G composites suggests these composites for application as actuators or electrical-energy-harvesting devices.

## 4. Conclusions

The results obtained in the present study show the control of the thermal, mechanical, and dielectric behavior in SEBS and SEBS-MA by using graphite as a filler. Here, control means the capacity of the G filler to influence and direct the properties or the course of their variation with temperature. A cumulative effect of grafted MA and graphite in improving the thermal and mechanical properties of SEBS was emphasized by TGA and DMA results. An important increase in mechanical stiffness with an increase in G content was mainly observed in the glassy region of PS blocks between 30 and 80 °C. The reinforcing of SEBS is due to the creation of flexible nets of polymer chains between G particles through the fixation of PS blocks on G particles by quadrupolar π-π interactions and to the interactions between the G and MA groups of SEBS-MA through hydrogen bonds between MA and surface carbonyl/carboxyl on G.

An exponential increase in ε_r_*’* with G content, with almost the same rate at 27 °C as at 77 °C, was also emphasized by the DS results for the SEBS or SEBS-MA composites. The exponential increase in ε_r_*’* with G concentration shows a possible control of dielectric permittivity by graphite concentration in composites, which is very useful in order to tailor dielectric elastomer composites for sensing, actuation, or energy-harvesting applications.

The ε_r_*’* variation with G is only slightly more rapid in the case of SEBS-MA composites compared to SEBS ones, which shows that the contribution of MA grafts to the new lateral dipoles is only marginal with respect to that of G flakes. However, the HF peak of the dielectric loss tangent is shifted to higher frequencies for SEBS-MA composites compared to SEBS/G composites, indicating stability in dielectric activity in a higher frequency range for composites with MA, which is very important for applications.

A low level of dielectric losses for both SEBS/G and SEBS-MA/G composites, as well as for unfilled SEBS and SEBS-MA polymers, was emphasized by the DS results, the tan δ having the order 10^−^^3^ for all the tested composites, up to hundreds of kHz. Moreover, the high stability of dielectric loss tangent tan δ with temperature variation was also remarked on for high G levels in SEBS/G and SEBS-MA/G composites. This stability was higher in the case of SEBS-MA/G composites, where the tan δ spectra at different temperatures were closer to each other than in the case of SEBS/G composites.

The HN fitting results of the imaginary part of the complex permittivity emphasized two dielectric relaxations as well as a DC conduction process. The activation energy corresponding to the MWS relaxation is practically the same for SEBS and in SEBS-MA neat polymers, whereas, for the composites, the activation energy increases with G content, with higher values for SEBS-MA/G than for SEBS/G composites, indicating reduced charge mobility, mainly due to the fixation of PS blocks on G flakes. As for the β-relaxation located at 10^5^–10^6^ Hz, besides the shifting of the loss peak towards higher frequencies for SEBS-MA composites than in the case of SEBS/G composites, the activation energy for this process is higher in MA-grafted SEBS composites.

This work shows the possibility of controlling and tailoring the dielectric properties of SEBS elastomers for specific applications, and it should be continued for even more significant improvement in dielectric permittivity.

## Figures and Tables

**Table 1:** Characteristic temperatures from DSC curves—heating and cooling scans.

Composites	*T_gPEB_*, °C I Heating	*T_mPEB_*, °C I Heating	*T_c_*,°C Cooling	Δ*H_c_*, J/g Cooling	*T_gPEB_*,°C II Heating	*T_mPEB_*, °C II Heating
SEBS	−57	20	4.7	3.6	−57	21
SEBS/G5	−57	21	5.5	3.6	−57	23
SEBS/G15	−56	18	4.6	3.3	−57	20
SEBS-MA	−56	23	3.9	3.7	−56	27
SEBS-MA/G5	−56	22	4.4	3.6	−56	27
SEBS-MA/G15	−57	18	4.3	2.9	−56	25

**Table 2:** Atomic concentration of different functional groups at the surface of the samples.

Composites	Composition (at%)			
	C1 (C sp2)	C2 (C sp3)	C3 (O-C-O/C=O)	C4 (C-O)
SEBS	17.0	82.1	0.9	-
SEBS/G20	32.1	64.9	0.5	2.5
SEBS-MA	16.6	81.9	1.5	-
SEBS-MA/G20	29.9	69.0	1.1	-

**Table 3:** Activation energies corresponding to MWS and β relaxations.

Composites	*w_a_* [eV]	
	MWS Relaxation (MF Peak)	β Relaxation (HF Peak)
SEBS	0.5306	0.0724
SEBS/G10	0.7331	0.1905
SEBS-MA	0.5783	0.103
SEBS-MA/G10	1.0512	0.4235

## Data Availability

The data presented in this study are available on request from the corresponding author.
